# Cytotoxicity and genotoxicity of bioceramic root canal sealers compared to conventional resin-based sealer

**DOI:** 10.1038/s41598-024-54726-1

**Published:** 2024-02-19

**Authors:** Mateusz Radwanski, Wioletta Rozpedek-Kaminska, Grzegorz Galita, Natalia Siwecka, Jerzy Sokolowski, Ireneusz Majsterek, Mutlu Özcan, Monika Lukomska-Szymanska

**Affiliations:** 1 Department of Endodontics, Medical University of Lodz, Lodz, Poland; 2 Department of Clinical Chemistry and Biochemistry, Medical University of Lodz, Lodz, Poland; 3 Department of General Dentistry, Medical University of Lodz, 251 Pomorska Str., 92-213 Lodz, Poland; 4 Clinic of Masticatory Disorders and Dental Biomaterials, Center for Dental Medicine, University of Zurich, Zurich, Switzerland

**Keywords:** Dental biomaterials, Dentistry, Dental materials, Endodontics

## Abstract

The aim of this study was to evaluate cytotoxicity and genotoxicity of calcium-silicate based sealers and comparing them with a gold standard—an epoxy-based sealant. Two experimental cell lines were used, gingival fibroblasts (hGF) and monocyte/macrophage peripheral blood cell line (SC). The cytotoxicity (XTT assay) and genotoxicity (comet assay) were evaluated both after 24-h and 48-h incubation. Additionally, after 48-h incubation, the cell apoptosis and cell cycle progression was detected. BioRoot Flow induced a significant decrease in hGF cells viability compared to the negative control groups both after 24-h (*p* < 0.001) and 48-h incubation (*p* < 0.01). In group with SC cells, after 24-h incubation significant increase in cells viability was detected for AH Plus Bioceramic Sealer in comparison to negative control (*p* < 0.05). BioRoot Flow and BioRoot RCS can be considered potentially genotoxic for the hGF cells after 48-h incubation (> 20% DNA damage). BioRoot Flow and BioRoot RCS, may have potential genotoxic effects and induce apoptosis in hGF cells which may irritate periapical tissues, resulting in a delayed healing. The findings of the study would be useful in selection of an appropriate sealant for root canal filling without causing cytotoxicity and genotoxicity.

## Introduction

Root canal filling is one of the most important stages of endodontic treatment. It should be homogeneous and non-resorbable in order to provide fluid-tight seal of canal space, and inhibit bacterial growth^1^. The root canal filling consists of a core, which is usually gutta-percha (GP), and a sealant^2^. Root canal sealers binding the core material to the canal walls, obturate the lateral canals and anastomoses, and fill spaces between cones used in the obturation techniques^3,4^.

There are many root canal sealers available on the market with different chemical compositions and setting reactions. According to their composition they can be classified into five main groups: zinc oxide eugenol (ZOE) based, calcium hydroxide based, glass ionomer-based, resin-based, and bioceramic sealers^1^.

AH Plus (Dentsply DeTrey, Konstanz, Germany) is a hydrophobic epoxy-resin sealant (resin-based) that exhibits long-term sealing integrity, low dimensional changes and high radiopacity^5^. It is widely used in dental practice therefore recommended as the gold standard^6–8^. However, some disadvantages of this material should be acknowledged, namely difficulties in connection to gutta-percha^9^, questionable cementation to the canal walls in the presence of moisture^10^, potential cytotoxicity and genotoxicity^11^ and difficulties in removal during retreatment^5^.

Calcium silicate-based sealers (CSBS, bioceramic) have become popular recently^12^. Different forms of CSBS were introduced as powder-liquid and premixed products. CBCS during setting hydration reaction release calcium and hydroxyl ions resulting in pH raise (> 12). As a consequence, these materials exhibit a long-lasting antibacterial effect^12–14^. Furthermore, bioceramic sealers may bond to the root canal dentine as a result of hydroxyapatite formation which combines chemically with dentinal tubules^15,16^. The main disadvantages of CSBS are difficulty in removal (during retreatment), especially at the apical third^16^, incompatibility with thermal methods^17^, resorption over time^12^ and potentially unfavourable interaction with rising solutions e.g. ethylenediaminetetraacetic acid (EDTA)^18^.

Among the many desirable features of sealants, one is biocompatibility. According to the International Organization for Standardization (ISO) 1942, the biocompatible material used in dentistry does not cause any adverse local or systemic effects when contact with vital tissue, but provides the most beneficial host response^19,20^. The toxicity of root canal sealers can be related directly to their components such as eugenol, bisphenol A, resin monomers; substances that are released during the setting reaction (e.g., formaldehyde) or after due to their solubility (e.g., calcium hydroxide). Although sealers are designed to remain inside the canal, due to the flow properties they may be unintentionally forced into the periapical tissues through the apical foramen or/and lateral and accessory canals^21,22^. Sealants in contact with tissue fluids may dissolve, thereby leading to leaching of components from them. The substances formed due to the degradation of sealants may be in long-term contact with periapical tissues and thus induce cytotoxic and genotoxic effects^23,24^. They can temporarily or permanently enter the bloodstream or come into contact with other tissue fluids, causing irritation, inflammation and possibly delayed healing after endodontic procedures^19,25^.

While there are many studies on the cytotoxicity of bioceramic sealers, there are only limited studies investigating their genotoxicity^6,26,27^. Additionally, most studies focused on dental-driven cell lines and thus analysed the local toxicity effect^28,29^. Hence, the study on the systemic impact of bioceramic sealers by selecting monocyte/macrophage peripheral blood cell line (SC) should be investigated. In particular, research on cytotoxicity and genotoxicity of BioRoot Flow (Septodont, Saint Maur Des Fosses, France) are missing. Thus, the aim of this in vitro study was to evaluate cytotoxicity and genotoxicity of calcium-silicate based sealers and comparing them with an epoxy-based sealant. The null hypothesis was that there would be no difference among the toxicity presented by the tested sealers.

## Materials and methods

### Root canal sealers

In the present study, five root canal sealers were analysed: one epoxy-resin based (AH Plus) and four calcium-silicate based sealers (TotalFill BC Sealer, AH Plus Bioceramic Sealer, BioRoot Flow and BioRoot RCS) (Table 1). All experiments were approved by the Committee of Ethics of the Medical University of Lodz, Poland (RNN/269/22/KE; 13/12/2022).

**Table 1:** Materials used in this study.

Root canal sealer	Manufacturer	Composition
AH Plus	Denstply DeTrey GmbH, Konstanz, Germany	Paste A: bisphenol-A epoxy resin, bisphenol-F epoxy resin, calcium tungstate, zirconium oxide, iron oxide, pigments Paste B: dibenyldiamine, aminoadamantane, tricyclodecane-diamine, calcium tungstate, zirconium oxide, silica, silicone oil
Total Fill BC Sealer	FKG Dentaire, La Chaux-de-Fonds, Switzerland	Calcium silicates, calcium phosphate monobasic, zirconium oxide, tantalum oxide, and thickening agents
AH Plus Bioceramic Sealer	Manufactured by Maruchi Distributed by Denstply DeTrey GmbH Konstanz, Germany	Zirconium dioxide (50–75%), tricalcium silicate (5–15%), dimethyl sulfoxide (10–30%), lithium carbonate (< 0.5%), thickening agent
BioRoot Flow	Septodont, Saint Maur Des Fosses, France	Tricalcium silicate, propylene glycol, povidone, calcium carbonate, aerosil (silica), zirconium oxide, acrylamide/sodium acryloyldimethyltaurate copolymer, isohexadecane and polysorbate
BioRoot RCS	Septodont, Saint Maur Des Fosses, France	Powder: Tricalcium silicate, zirconium oxide, and povidone Liquid: Aqueous solution of calcium chloride and polycarboxylate

### Cell lines and elute preparation

All the in vitro analyses were performed using two experimental cell model lines: human gingival fibroblasts (ATCC CRL-2014) (ATCC; Manassas, VA, USA) and monocyte/macrophage peripheral blood cell line—SC (ATCC CRL-9855) (ATCC; Manassas, VA, USA).

#### Human gingival fibroblasts cell line (hGF-1)

Cell cultures were maintained under standard conditions (37 °C; 5% pCO2; 95% humidity) according to the manufacturer guidelines. Cells were cultured in Dulbecco Modified Eagle Medium (DMEM) with fetal bovine serum (10%) (ATCC; Manassas, VA, USA) and 1% penicillin/streptomycin (P/S) solution (ScienCell Research Laboratories, advertising San Diego, CA, USA). Cells were passaged when the culture reached 90–95% confluency.

#### Monocyte/macrophage peripheral blood cell line (stem cells; SC)

Cell cultures were maintained under standard conditions (37 °C; 5% pCO_2_; 95% humidity) according to the manufacturer guidelines. Cells were cultured in Iscove's Modified Dulbecco's Medium (IMDM) with 4-mML-glutamine adjusted to 1.5 g/L sodium bicarbonate (ATCC; Manassas, VA, USA) and supplemented with 0.05 mM 2-mercaptoethanol (Sigma- Aldrich Corp., St. Louis, MO, USA), 0.1 mM hypoxanthine and 0.016 mM thymidine (90%) (ATCC; Manassas, VA, USA), fetal bovine serum (10%) (ATCC; Manassas, VA, USA) and 1% penicillin/streptomycin (P/S) solution (ScienCell Research Laboratories, advertising San Diego, CA, USA). Cells were divided when the culture reached 90–95% maturity.

#### Elute preparation

The sealants were prepared in accordance with the manufacturers’ recommendations. For the cytotoxicity and genotoxicity tests, 1.4 g of each sealant was use and for apoptosis and cycle analysis, 0.7 g were spread on the lower surface of a sterile 50 mL centrifuge tube. After setting time, sealants were covered with 3.5 mL of culture medium and eluted for 24 h in a humidified atmosphere containing 5% CO_2_. After this time, 5 mL was collected from each tube and filtered to remove particulate matter. These elutes were then diluted tenfold in culture medium to bring it into contact with the cultured cells.

### Cytotoxicity analysis

The cytotoxicity of the sealers was measured using a colorimetric, resazurin-based assay kit (Sigma Aldrich Corp., St. Louis, MO, USA). The mechanism is based on the irreversible reduction of resazurin by dehydrogenase enzymes to pink and bright red fluorescent resorufin only in metabolically active cells. The whole experiments were performed in triplicate with similar results. SC and hGF cells were seeded in 96 well plates (8 × 10^3^/well) and after 24 h investigated compounds were added at the appropriate concentrations. Untreated cells cultured in a complete medium were used as a negative control, whereas cells incubated with 100% dimethyl sulfoxide (DMSO) comprised a positive control. Cells were incubated for 24 h and 48 h at 37 °C, respectively. Then, the cell plates were centrifuged and 100 μL of a 10% resazurin solution was added to each well. After 4-h incubation at 37 °C absorbance at 600 nm and a reference wavelength of 690 nm was measured with a Synergy HT spectrophotometer (BioTek, Vermont, VT, USA)^30,31^.

### Genotoxicity assessment

The alkaline version of the comet assay to analyse deoxyribonucleic acid (DNA) damage in specific cells was used for genotoxicity evaluation of the tested materials. Assays were prepared in 12-well plates by adding 5 × 10^4^ cells/well and after 24 h test compounds were added at the appropriate concentrations. Cells preserved in highly toxic 10% DMSO (Sigma-Aldrich Corp., St. Louis, MO, USA) was a positive control, whilst cells suspended in 1 mL of complete culture medium constituted a negative control. The specimens were incubated for 24 h and 48 h. Cells suspended in 0.37% low melting point (LMP) agarose (Sigma-Aldrich Corp., St. Louis, MO, USA) were placed on microscope slides that were previously coated with normal melting point (NMP) agarose (Sigma-Aldrich Corp., St. Louis, MO, USA). Preparations were incubated in lysis buffer at pH 10 (2.5-M NaCl, 10-mM Tris, 100-mM EDTA), containing TritonX-100 (Sigma-Aldrich Corp., St. Louis, MO, USA), at a final concentration of 1% at 4 °C for 60 min. After 1-h incubation, the specimens were incubated in development buffer (300-mM NaOH, 1-mM EDTA) for 20 min at 4 °C and this was followed by electrophoresis (32 mA, 17 V, 20 min) at 4 °C in electrophoretic buffer (30-mMNaOH, 1-mM EDTA). Finally, 4′,6-Diamidino-2-phenylindole (DAPI) was used for staining, and the obtained data were evaluated under a fluorescence microscope by assessing the percentage of DNA in the comet tail^30,31^.

### Apoptosis detection

The fluorescein isothiocyanate FITC Annexin V Apoptosis Detection Kit I (FITC Annexin V Apoptosis Detection Kit I, BD Bioscences, NJ, USA) was used to assess apoptotic cell death. Assays were prepared in 12-well plates by adding 1 × 10^6^ cells/well and after 24 h test compounds were added at the appropriate concentrations and incubated for 48 h. The positive control comprised cells treated for 16 h with 1 μM staurosporine (Sigma-Aldrich Corp., St. Louis, MO, USA), while negative control represented cells incubated for 48 h in complete culture medium. Afterwards, the cells were washed twice with cold phosphate-buffered saline (PBS) (Sigma-Aldrich Corp., St. Louis, MO, USA) and then double-stained with: annexin V (an early apoptosis marker) and propidium iodide (PI) (cell membrane disintegration, necrosis, and late apoptosis marker). CytoFLEX (Beckman Coulter, Brea, CA, USA) was used to calculate the percentage of apoptotic cells by flow cytometry (FC) and the obtained data were analysed using Kaluza Analysis 1.5 A (Beckman Coulter, Brea, CA, USA)^30,31^.

### Cell cycle analysis

The analysis of the cell cycle was performed by FC using PI staining. Assays were prepared in 12-well plates by adding 1 × 10^6^ cells/well and after 24 h tested compounds were added at the appropriate concentrations and incubated for 48 h. The positive control constituted cells treated for 16 h with 1 μM nocodazole (Sigma-Aldrich Corp., St. Louis, MO, USA), whereas cells cultured in a complete medium for 48 h constituted a negative control. Afterwards, cells were washed twice with cold PBS (Sigma-Aldrich Corp., St. Louis, MO, USA) and then preserved with ice-cold 70% ethanol at − 20 °C for 20 min. Before staining with PI solution (10 μg/mL) (Sigma-Aldrich Corp., St. Louis, MO, USA) cells were processed with RNase A DNase & Protease-free (10 mg/mL) (Canvax Biotech, Córdoba, Spain) and incubated at 37 °C for 1 h. The percentage of cells in each phase of the cell cycle was assessed using Kaluza Analysis 1.5A software (Beckman Coulter, Brea, CA, USA). In the DNA content histograms, the x-axis showed the DNA content measured by PI fluorescence, while the y-axis indicated the number of cells^30,31^.

### Statistical analysis

All statistical analyses were evaluated with the statistical software package Statistica 13 (StatSoft, Kraków, Poland). The Shapiro–Wilk test was used to confirm the normality of the data. The Student’s t-test was used for paired normally distributed samples comparison, otherwise the Mann–Whitney rank sum test was performed. All analyses in each experiment were based on the results of three independent tests. Statistically significant differences are presented in the graphs as follows: *p < 0.05; **p < 0.01; ***p < 0.001 compared to negative control.

## Results

### Analysis of the cytotoxicity of the sealers

**Figure 1:**
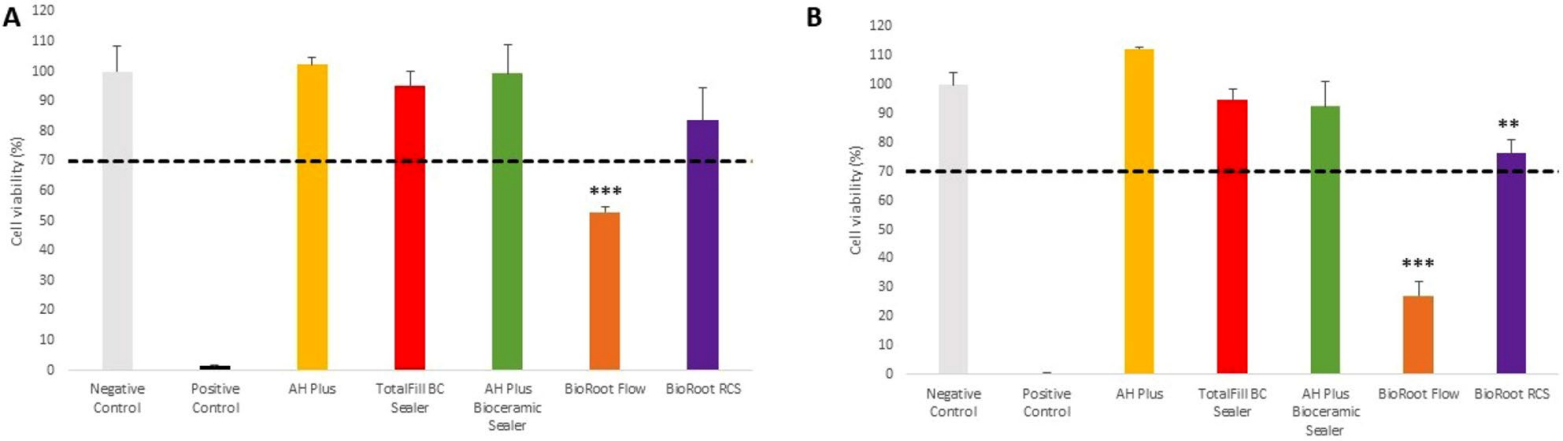
The cell viability assay after 24-h (**A**) and 48-h incubation (**B**) of hGFcells with root canal sealers. Statistical significance on the graphs: *p < 0.05; **p < 0.01; ***p < 0.001 versus negative controls. The dashed lines represent ISO 10993 cut off levels (70%).

The cytotoxicity analysis showed that BioRoot Flow induced a significant decrease in hGF cells viability compared to the negative control groups both after 24-h and 48-h of incubation (Fig. 1A,B) (*p* < 0.001). Moreover, BioRoot RCS significantly reduced number of vital hGF cells after 48 h (*p* < 0.01) when compared with negative control.

**Figure 2:**
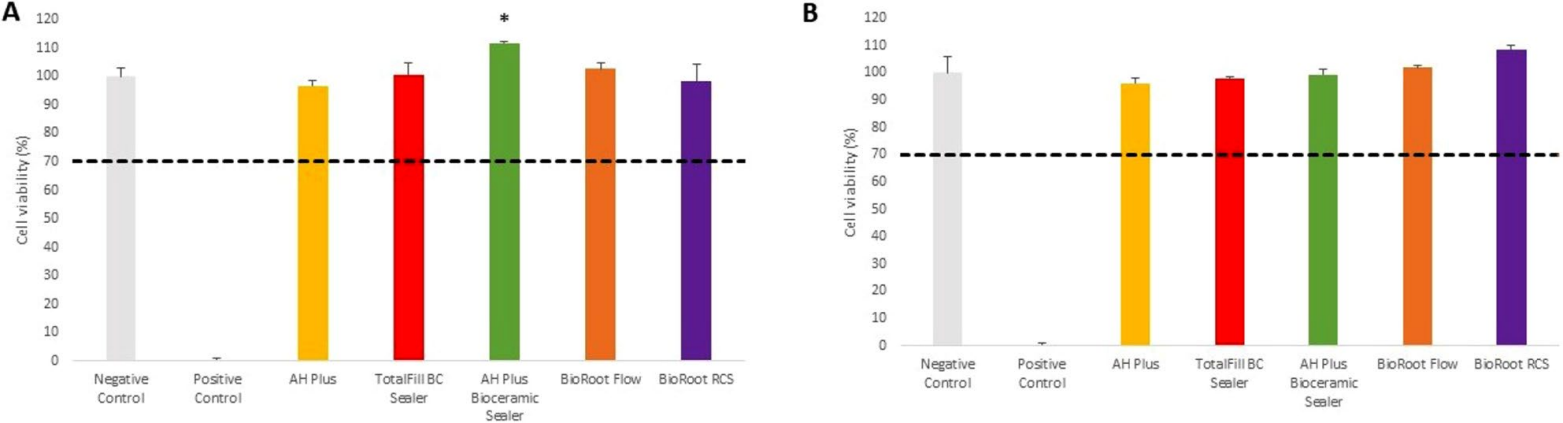
The cell viability assay after 24-h (**A**) and 48-h incubation (**B**) of SC cells with root canal sealers. Statistical significance on the graphs: *p < 0.05; **p < 0.01; ***p < 0.001 versus negative controls. The dashed lines represent ISO 10993 cut off levels (70%).

In group with SC cells, after 24-h incubation the significant increase in cells viability was detected for AH Plus Bioceramic Sealer in comparison with negative control (*p* < 0.05) (Fig. 2A,B).

### Analysis of the genotoxicity of sealers

**Figure 3:**
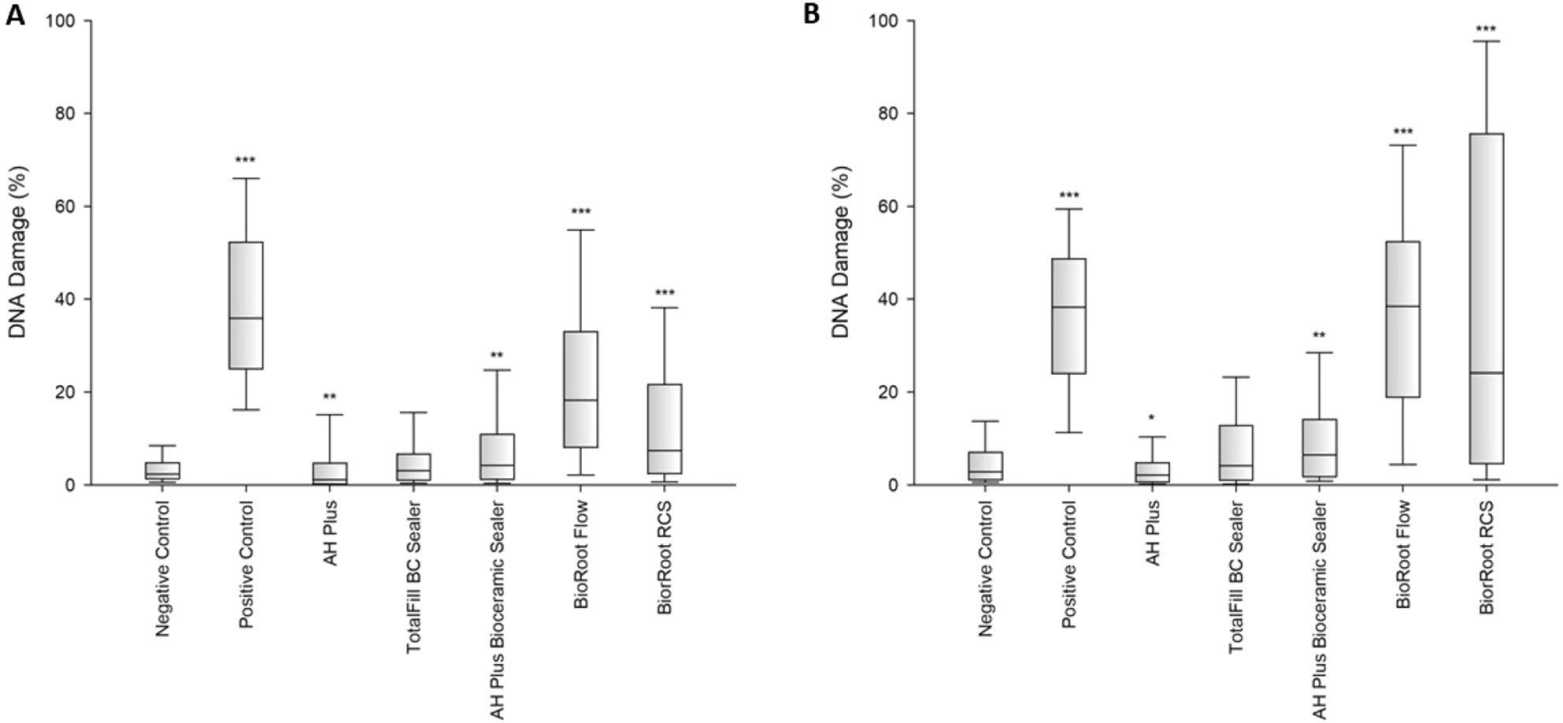
Genotoxicity after 24-h (**A**) and 48-h (**B**) incubation of hGF cells with the tested compounds. Statistical significance on the graphs: *p < 0.05; **p < 0.01; ***p < 0.001 versus negative controls.

**Figure 4:**
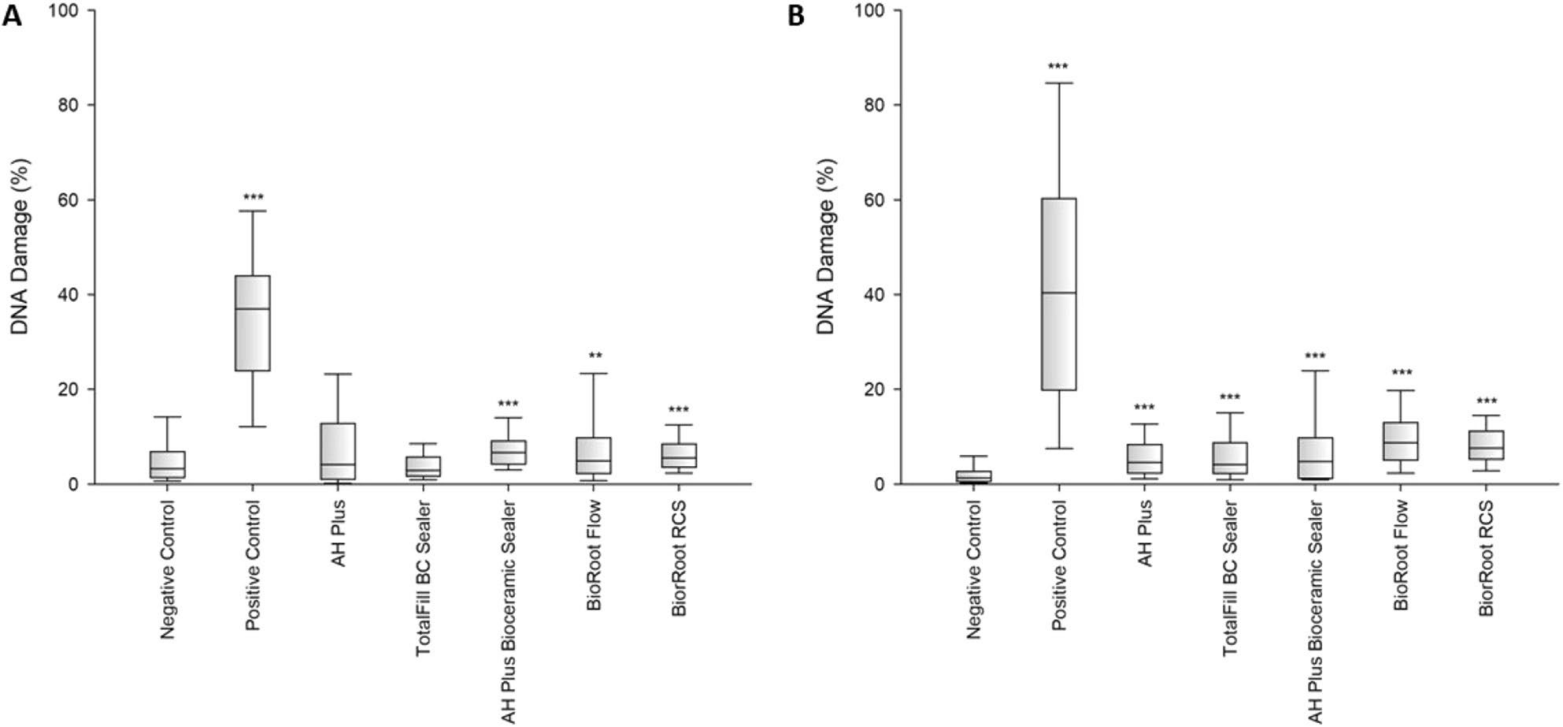
Genotoxicity after 24-h (**A**) and 48-h (**B**) incubation of SC cells with the tested compounds. Statistical significance on the graphs: *p < 0.05; **p < 0.01; ***p < 0.001 versus negative controls.

Considering the hGF cell line, both after 24-h and 48-h statistically significant higher DNA damage compared to the negative control was observed for all sealers except for TotalFill BC Sealer (Fig. 3). In case of the SC cell line, after 24-h incubation, DNA damage compared to the negative control was statistically significantly higher for all sealers except for AH Plus and TotalFill BC Sealer (Fig. 3). With a 48-h incubation time for this line, statistically higher DNA damage was found for all evaluated sealers (Fig. 4) compared to negative control.

### Apoptosis detection

**Figure 5:**
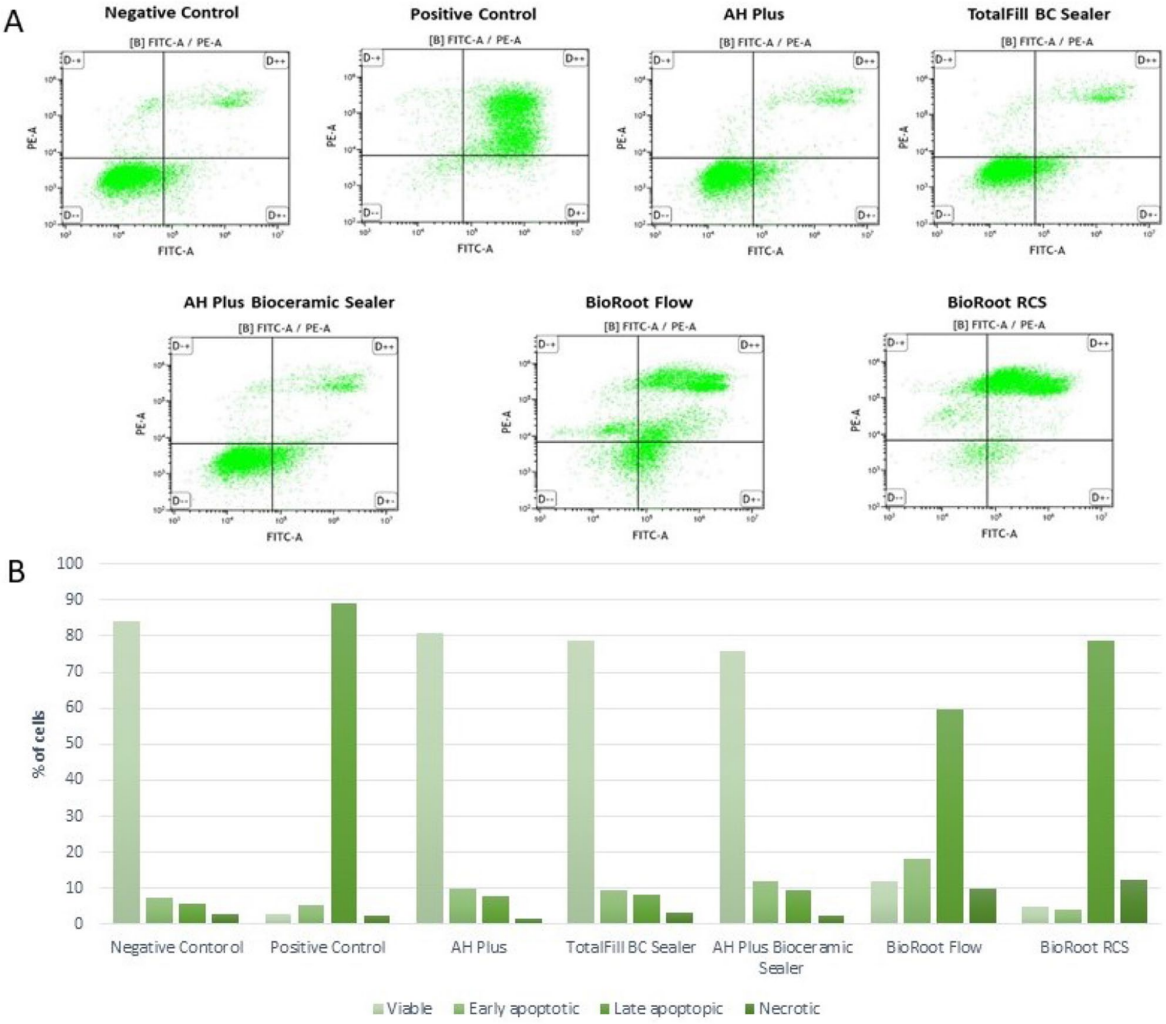
Dot plot graphs of flow cytometric FITC annexin V/propidium iodide (PI) double staining analysis of apoptosis after 48-h incubation of hGF cells with the tested compounds (**A**). Percentage of viable, early, and late apoptotic, and necrotic cells in each group (**B**).

**Figure 6:**
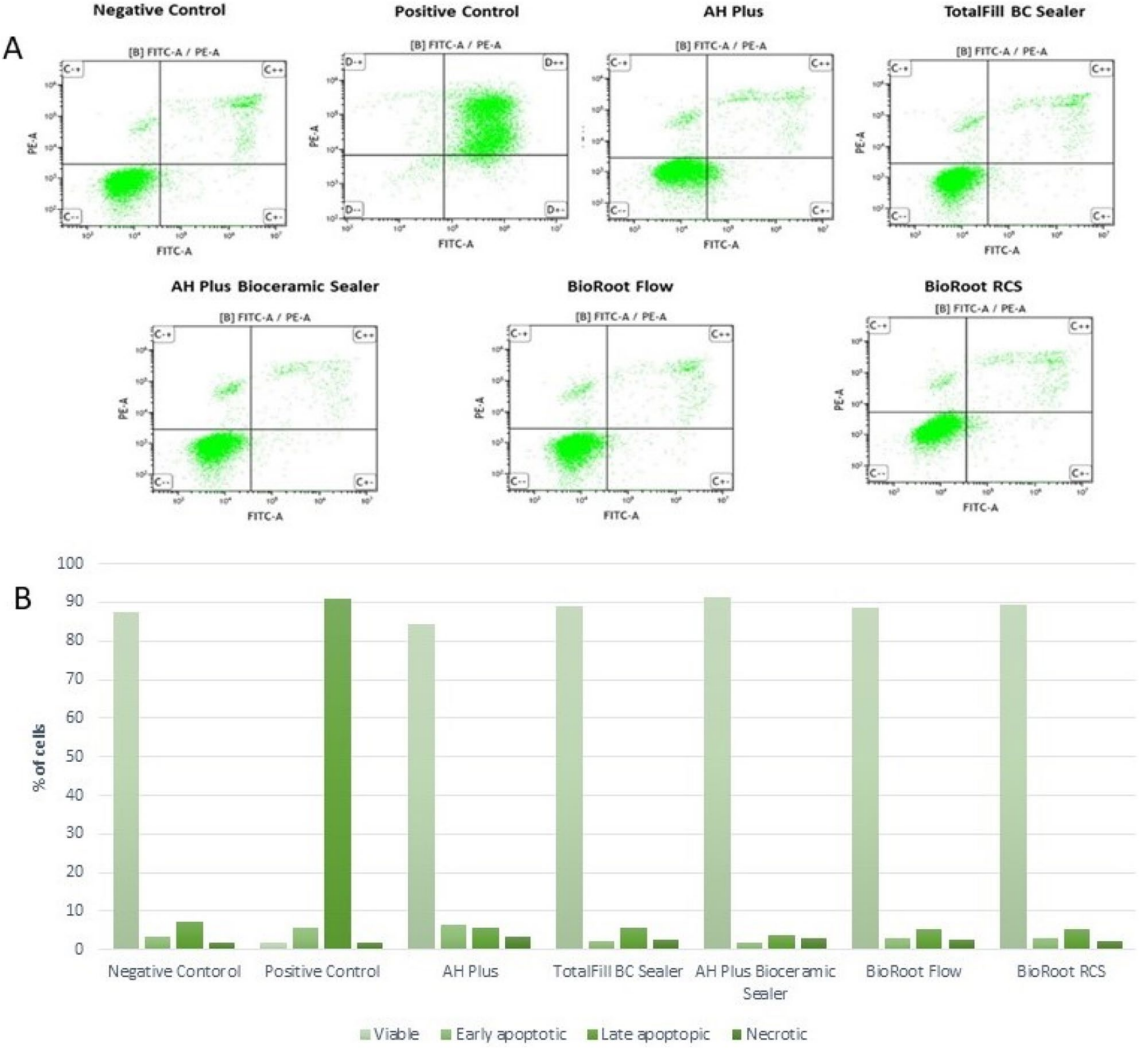
Dot plot graphs of flow cytometric FITC annexin V/propidium iodide (PI) double staining analysis of apoptosis after 48-h incubation of SC cells with the tested compounds (**A**). Percentage of viable, early, and late apoptotic, and necrotic cells in each group (**B**).

After 48-h incubation, BioRoot Flow and BioRoot RCS significantly induced apoptosis in hGF group; ca. 78% and 83% of cells were at the early and late stages of apoptosis, respectively (Fig. 5). Additionally, none of the tested compounds evoked a significant increase in the level of apoptotic and necrotic SC cells (Fig. 6) (*p* > 0.05).

### Analysis of the cell cycle progression

**Figure 7:**
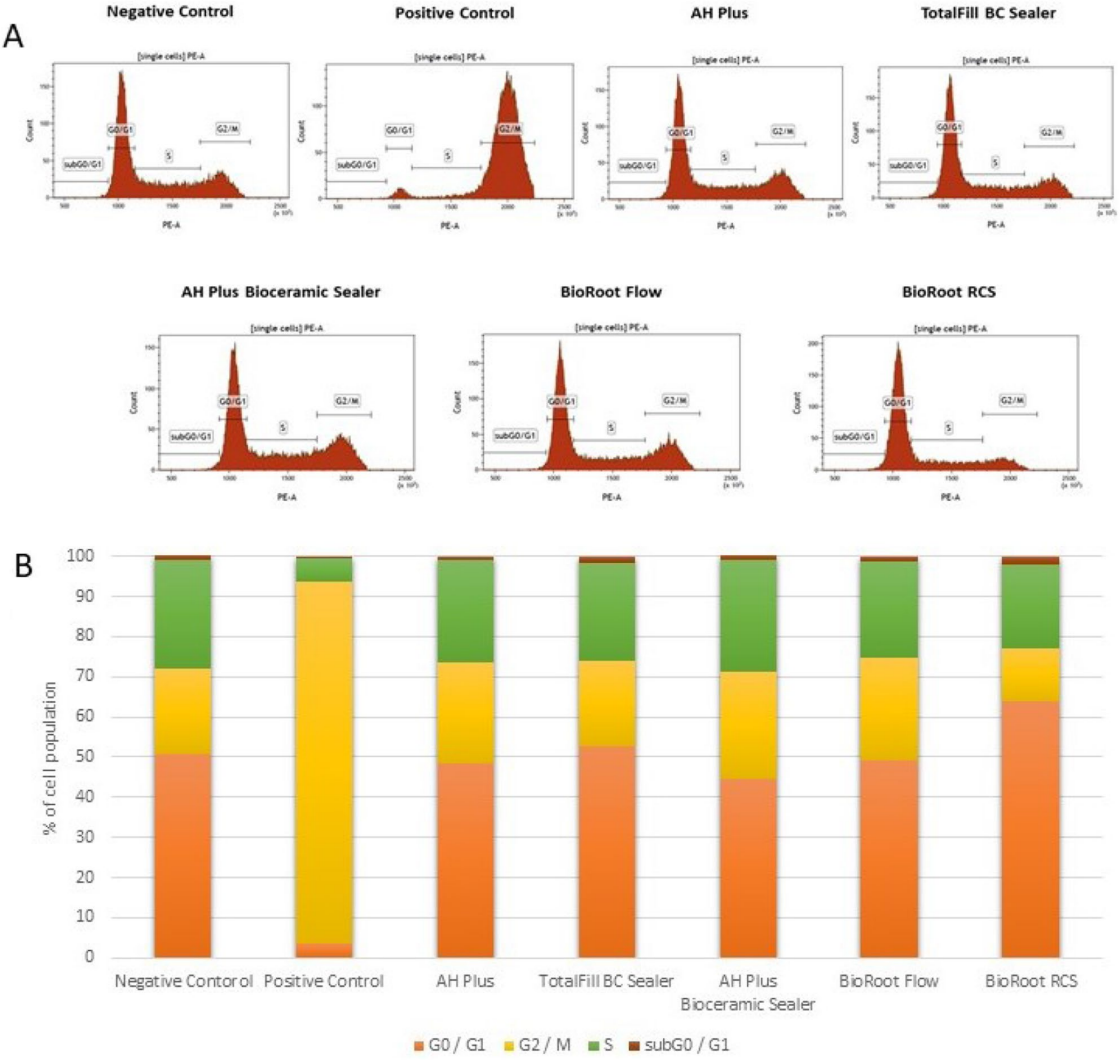
Flow cytometry (FC) analysis of cell cycle progression using propidium iodide (PI) staining after 48-h incubation of hGF cells with the tested compounds (**A**). Percentage of cells in different cell cycle stadium (**B**).

**Figure 8:**
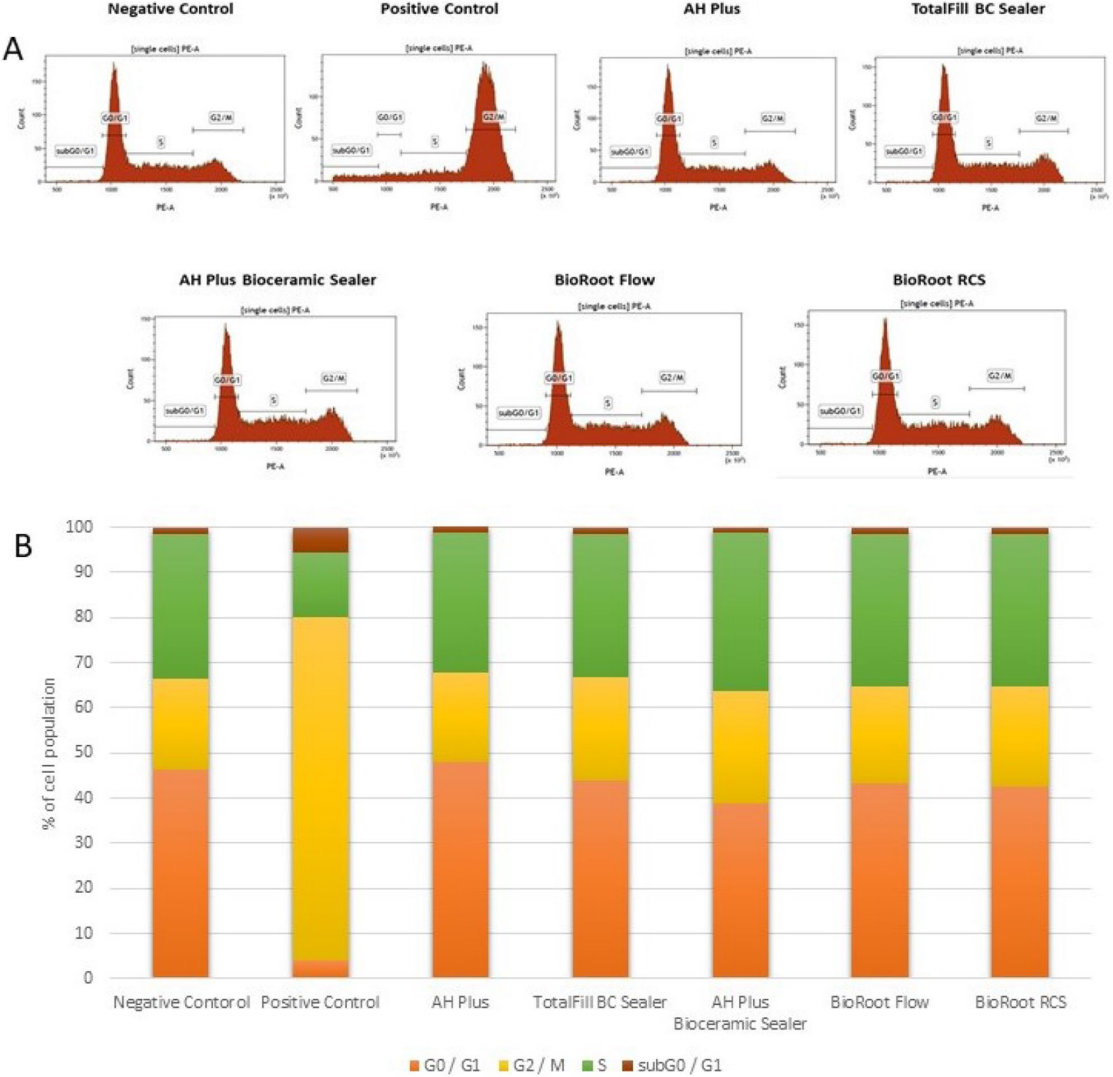
Flow cytometry (FC) analysis of cell cycle progression using propidium iodide (PI) staining after 48-h incubation of SC cells with the tested compounds (**A**). Percentage of cells in different cell cycle stadium (**B**).

After 48-h incubation, the cell cycle progression of the hGF and SC cells treated with all tested root canal sealers was similar to the cells cultured in the complete medium (*p* > 0.05) (Figs. 7, 8).

## Discussion

Root canal sealers should be biocompatible since they can be in direct contact with the periapical tissues (in case of material extrusion) or indirectly through the products released during the setting reaction^32^. Therefore, before employment of the material in clinical practice, potential cytotoxicity and genotoxicity should be verified^21^. In the present study the null hypothesis was rejected, because statistically significant differences in cytotoxicity and genotoxicity were found between tested sealers.

It is worth highlighting that no studies, to date, have evaluated the biocompatibility of endodontic sealers using multiple in vitro tests as presented in this study. Furthermore, two cell lines were used, which provided the assessment of both local and systemic effects of the tested materials. What is more, the results of the study may be helpful in choosing the appropriate material in endodontic treatment.

In the present study, the endodontics sealers were tested after the initial setting of 24 h and 48 h to simulate what happens clinically 1 and 2 days after root canal system obturation. Therefore, the toxicity of the tested materials was related to the components released after hardening or the result of irritation by the substances not reacted during the setting reaction.

### Cytotoxicity of root canal sealers

AH Plus was the most widely studied in cytotoxicity assessment, thus in our study was considered as the reference sealer^19,33–38^. In the present study, set AH Plus exhibited above 90% of viable hGF and SC cells after both incubation periods. Thus, it can be regarded as non-cytotoxic, since according to ISO 10993 standards, a sealer is cytotoxic if cell viability is below 70%^34,35^. This result is in accordance with previous studies^39–41^. In contrast, some studies indicated cytotoxicity of this sealer^34,35^, especially when freshly mixed (non-hardening)^19,36^. The release of small amount of formaldehyde or bisphenol-A during setting reaction of the epoxy resin, were indicated as a potential cytotoxic substance in AH Plus composition^34,35,37^. On the contrary, some studies reported stronger toxicity of the set sealer^38,40^. The discrepancies between studies may be explained by different experimental conditions including sample preparation, exposure time and cell cultures (2D or 3D). In 3D cell aggregates, there is a stronger interaction between the cells and the matrix when compared to 2D cell culture, thus reduced ability to penetrate the sealant extracts which results in lower cytotoxic effect^40^. Most studies confirmed that toxicity of this sealant decreased with observation time and might persist even up to 2 weeks^19,25,42,43^.

Numerous studies suggested that CSBS sealers may present lower cytotoxic potential contrasted to other types of root canal sealers^25,44–48^. In the case of bioceramic sealants, the cytotoxic effect may be related to the release of substances during the setting and subsequent dissolution of the material (calcium ions)^40^, the addition of radiopacifiers or thickening agents^49^, the content of oxides, mainly barium oxide^50^, and the formation of hydroxyapatite^49^.

In the present study only the BioRoot Flow can be considered as locally moderately cytotoxic (30–59%), as it significantly reduced only hGF cells viability compared to negative control and the cytotoxic effect increased after 48-h incubation. It should be emphasized that up to date in the literature there have been no studies on BioRoot Flow; presumably the local cytotoxicity of this material may be due to some components such as acrylamide which is a thickener and flocculating agent. Studies confirmed its cytotoxicity against different cell lines (HEK293, A549), which increases with higher concentrations and longer exposure time^51,52^.

The other tested CSBS sealants met ISO cytotoxicity criterium (> 70% viable cells)^34,35^. BioRoot RCS was reported as less toxic compared to zinc-oxide materials^44,45,47^ or resin-based sealer (AH Plus)^48,53^. Contrary, in the present study this material exhibited higher cytotoxicity than AH Plus against hGF (local cytotoxicity). In addition, one study reported moderate-slight cytotoxicity (57.51%) of BioRoot RCS to NIH 3T3 fibroblasts cells^37^. Non-cytotoxic effect of TotalFill BC Sealer was confirmed by others which is in accordance with the present results^46^. Additionally, TotalFill BC Sealer exhibited less cytotoxicity than freshly mixed resin-based sealer (AH Plus)^48^ and zinc-oxide sealer (Pulp Canal Sealer; Kerr, Romulus, MI, USA)^54^. In contrast, one study showed higher cytotoxicity of TotalFill BC Sealer when compared to AH Plus (set material)^54^. Moreover, AH Plus Bioceramic Sealer was reported less cytotoxic compared to AH Plus^34,35^; however, in the present study both materials exhibited no cytotoxicity (> 90%) against tested cells. In addition, in the group of SC cells after 24 h, AH Plus Bioceramic Sealer significantly increased cell viability (~ 112%), which may indicate the ability of this material to stimulate cell proliferation^15^. It may be hypothesised that this phenomenon facilitates tissue regeneration.

### Genotoxicity of root canal sealers

The genotoxicity tests were carried out to verify the effects of tested material on the genetic material of cells, which may affect their integrity^26^. Bankoglu et al. reported that DNA damage above 20% negatively affected cell viability, therefore, for in vitro experiments where this level of damage was found, it is recommended to provide cell survival data in parallel^55^. Accordingly, to this recommendation, in the present study, despite many statistically significant differences between the sealants and the negative control, only BioRoot Flow and BioRoot RCS can be considered potentially genotoxic for the hGF cells after 48-h incubation. It should be mentioned that it is the first study analysing the genotoxicity of BioRoot Flow, so direct comparison of results was not possible. It might be hypothesised that the genotoxicity of BioRoot Flow is caused by a sealer component—acrylamide. This chemical compound can be transformed to a more active metabolite, glycidamide, which increases genotoxicity^51,56,57^. In addition, research confirmed its effect on genome destabilization (e.g., nuclear condensations, fragmentations)^52^ and oxidative stress^51^. In the case of BioRoot RCS, genotoxicity may result from incomplete setting of the material, which is mixed manually. BioRoot RCS in one study showed the lowest genotoxic potential, although the higher concentration of this sealer showed negligible double-strand break formation thus may be considered as potentially genotoxic^58^.

There was no consensus in the literature regarding the genotoxicity of resin-based materials^19,58–60^. Some studies concluded that AH Plus is significantly more genotoxic than other sealers^19,59^, while others reported no genotoxicity which was in accordance with present results^58,60^. The unset epoxy-resin sealants were found to be more genotoxic than set material^19^. The genotoxicity of AH Plus was associated with the release of resin monomers, which enhance the formation of reactive oxygen species (ROS)^61^. As a consequence, the level of protective enzymes is disrupted, thus contributing to DNA damage and apoptosis^61^. Additionally, bisphenol-A and formaldehyde are widely considered as carcinogens and may promote genome instability^61–63^.

Studies investigating genotoxic potential of CSBS present inconclusive results^27,58,59,61^. Calcium silicate materials were claimed to exhibit the lowest genotoxicity potential when compared with other sealers group^61^, while others found TotalFill BC Sealer to be genotoxic^58^, but less than zinc oxide sealant (L929 cells)^27^ and AH Plus (FMM1 cells)^59^. Contrary, in our study, TotalFill BC Sealer was not statistically genotoxic for both tested cell lines regardless of the observation time.

### Cells apoptosis

After the necrosis cell content is released into the adjacent tissues, that stimulates immune cells to release enzymes and reactive oxygen, sustaining the inflammatory process; thereby causing a delayed healing^40,64^. In the present study, gingival fibroblast necrosis was mainly induced by BioRoot Flow and BioRoot RCS; ca 78% and 83% of cells were at the early and late stages of apoptosis, respectively. The assay for apoptosis authenticated results obtained in the cytotoxicity and genotoxicity assays. Hence, these results were in disagreement with previous study, in which BioRoot RCS revealed more than 87% of viable cells after exposure^65^.

In a study evaluating AH Plus, the authors detected 85.45% apoptotic cells in early and late-stage^40^ which was in contrary with the obtained results in the present study: > 80% of viable cells in both cell lines. In addition, one study showed that the highest cell death was observed at high concentrations of tested calcium silicate-based sealers (MTA Fillapex; Angelus Indústria de Produtos Odontológicos S/A, Londrina, PR, Brazil and TotallFill BC Sealer)^46^.

### Cells progression cycle and models

The cell cycle progression analysis showed not statistically significant changes compared to the negative control in case of the tested sealants. It is worth emphasising there are no studies on the cell cycle available in the literature, therefore a direct comparison of the results was not possible. It should be noted that any discrepancies between our results when compared with other in vitro studies could be related to methodological differences, such as materials setting conditions (whether materials were freshly mixed or set), sealer concentration (dissoluted or not), exposure time, cell type, and cytotoxicity and genotoxicity assays used. Various methods are used to assess the biocompatibility in vitro including cytotoxicity, genotoxicity tests, apoptosis detection and/or cell cycle progression^30,31^. Most studies apply test of cytotoxicity based on reduction of tetrazolium salts with different cell models with accordance to ISO standard 10993-5: 1999^21,66^. The advantage of these methods include simplicity, speed, precision, and reproducibility^27^. Moreover, the dental materials may induce genome instability thus comet assay method can be used for the quantitative DNA damage assessment (ISO10993-3). The test is very sensitive and detects the smallest level of DNA damage and requires a short time to perform at minimal cost and a limited number of cells per sample. The apoptosis, necrosis and cell cycle analysis are usually indirectly detected via the flow cytometry (FC)^67^. This method uses impermeable fluorescent dyes, such as propidium iodide (PI) capable of binding and labelling DNA fragments thereby providing a rapid and precise evaluation^67^.

In the literature different cell models for cell viability assessment were used including human cells, namely: gingival fibroblasts^37,68^, dental pulp stem cells^69^, osteoblasts^53,70^, periodontal ligament cells^43,47^ or human osteoblast-like cells (MG63)^71^, and non-human cells e.g.: L929 mouse fibroblasts^71,72^ or Chinese hamster fibroblasts (V79)^21^. In dental materials science, human cell lines derived from oral tissues were preferred for cytotoxicity and genotoxicity evaluation^73^. Fibroblasts are the main components of connective tissue and the dominant type of periodontal ligament cells that will contact endodontic sealers^37^. For that reason, immortalized human gingival fibroblasts (hGF) were used in this study because they can be cultured in a small number of passages, resulting in minimal cellular changes due to cell culture manipulation^38^. Beside the local effect, the assessment of systemic toxicity is very important, which, according to the PN-EN ISO 10993-11: 2018 standard, should be assessed on monocytes/macrophages as a stage of preclinical testing of biological materials, therefore second cell line (SC) was chosen for this experimental study^30^.

### Limitations and future perspective

Some limitations of the present study should be acknowledged. Firstly, the in vitro character of the study makes it impossible to assess the long-term effects occurring in human body. The study does not include some important factors e.g., host defence, which can prevent the toxicity of materials and thus protect against disintegration or disruption of the genome of cells. Further studies should include the development of a model to evaluate root canal sealers using a blood flow system that attenuates the toxic effects of dental materials. In addition, periapical or osteoblast cell lines were not used on this study which can be considered as a limitation and should be further investigated in future studies. Also, less commonly used cells e.g. neuronal cells can be used in the tests and brought in contact with sealants limited to an apical area. With the extensive demand for bioceramic materials, longitudinal and multicentre clinical trials are essential to provide a broader view of their features to clinicians. Moreover, different mixing techniques of investigated materials may also contribute to heterogeneity of findings. In other words, BioRoot RCS demands manual measurement and mixing of product components in contrast to other tested bioceramic sealers that are ready to use. Although this is the first study to evaluate the biocompatibility of BioRoot Flow, more in vitro and in vivo investigations are needed to verify and better understand the current findings. It is recommended that further research be undertaken using both traditional microscopic and spectroscopic techniques. Confocal laser scanning microscopy (CLSM), transmission electron microscopy (TEM) along with scanning electron microscopy (SEM) can help to investigate surface topography and visualise rapid chemical and biochemical properties of bioceramic sealers^74^. The above-mentioned techniques could be supplemented by atomic force microscopy (AFM), particularly when the surface roughness and nanomechanical properties in natural conditions (i.e. body fluids) should be investigated^74^. Moreover, spectroscopic methods such as Fourier Transform Infrared Spectroscopy (FT-IR) and X-ray Spectroscopy would help to further analyse these materials^75^. The former could provide more valuable data on microstructural and surface properties and the setting reactions of bioceramic sealers whereas X-ray Photoelectron Spectroscopy (XPS) and X-ray diffraction (XRD) would give an insight into the elemental compositions and the degree of crystallinity of these materials^75^.

Nevertheless, the correct choice of endodontic materials including sealers is essential for general health. It must be noted that when they are released from root apex or lateral and accessory canals, the leaching components may induce cytotoxic and genotoxic effects as they can enter the bloodstream. The consequences of such an effect on the infection development or neuronal cells needs to be further investigated.

## Conclusions

From this study, the following could be concluded:

1. AH Plus Bioceramic Selaer enhanced the viability of the SC cells after 24-h incubation.
2. BioRoot Flow exhibited moderate cytotoxicity locally.
3. BioRoot Flow and BioRoot RCS exhibited potential genotoxicity for the hGF cells after 48-h incubation.

## Data Availability

The datasets used and/or analysed during the current study available from the corresponding author on reasonable request.
